# Somatosensory-Motor Adaptation of Orofacial Actions in Posterior Parietal and Ventral Premotor Cortices

**DOI:** 10.1371/journal.pone.0049117

**Published:** 2012-11-20

**Authors:** Krystyna Grabski, Laurent Lamalle, Marc Sato

**Affiliations:** 1 Gipsa-Lab, Département Parole & Cognition, UMR CNRS 5216 & Grenoble Université, Grenoble, France; 2 INSERM; Structure Fédérative de Recherche N°1 “RMN Biomédicale et Neurosciences” & Unité IRM 3T, Centre Hospitalier Universitaire de Grenoble, Grenoble, France; Northwestern University, United States of America

## Abstract

Recent studies have provided evidence for sensory-motor adaptive changes and action goal coding of visually guided manual action in premotor and posterior parietal cortices. To extend these results to orofacial actions, devoid of auditory and visual feedback, we used a repetition suppression paradigm while measuring neural activity with functional magnetic resonance imaging during repeated intransitive and silent lip, jaw and tongue movements. In the motor domain, this paradigm refers to decreased activity in specific neural populations due to repeated motor acts and has been proposed to reflect sensory-motor adaptation. Orofacial movements activated a set of largely overlapping, common brain areas forming a core neural network classically involved in orofacial motor control. Crucially, suppressed neural responses during repeated orofacial actions were specifically observed in the left ventral premotor cortex, the intraparietal sulcus, the inferior parietal lobule and the superior parietal lobule. Since no visual and auditory feedback were provided during orofacial actions, these results suggest somatosensory-motor adaptive control of intransitive and silent orofacial actions in these premotor and parietal regions.

## Introduction

Single-unit neurophysiological recordings in nonhuman primates have provided evidence for movement selectivity and action goal coding of visually guided transitive manual motor acts (i.e., object-directed) in posterior parietal and ventral premotor areas [1], [2], [3], [4], [5], [6], [7]. Notably, grasping neurons in inferior parietal and ventral premotor areas have been shown to discharge differently according to the ultimate action goal (e.g., eating or placing the object) despite similar grasping movements [2], [6]. In humans, functional magnetic resonance imaging (fMRI) has been recently used in conjunction with adaptation to decode action goal coding of transitive and intransitive manual behaviours [8], [9], [10], [11], [12]. fMRI-adaptation, or repetition suppression effect (RS), refers to the phenomenon that repeated stimulus presentation or motor acts leads to a reduction in blood oxygen level-dependent (BOLD) signal in brain areas that are sensitive to the performed or observed action [13], [14]. In accordance with nonhuman primate studies, this approach has revealed that repeated manual actions with similar goals cause RS in the intraparietal sulcus and the surrounding dorsal part of the inferior parietal lobule, as well as in the inferior frontal gyrus and the adjacent ventral premotor cortex [8], [10], [11], [15], [16].

Although largely discussed in terms of action goal coding in the above-mentioned studies, RS has been proposed to reflect possibly enhanced adaptive updates/learning and increased processing efficiency in specific neural populations [13], [14]. From this view, a convergent computational interpretation of RS is based on forward sensory-motor adaptive control [17], [18]. A forward model is part of a generative model that associates actions with sensory consequences in which predictive coding schemes compare top-down predictions with bottom-up sensory information to create a prediction error for online state estimation and motor correction [17], [18], [19]. According to forward internal models [20], [21], [22], predicted sensory consequences of a motor act, or sensory goals, are generated by means of an efference copy in parallel with the motor command. In case of discrepancy between the prediction and the actual sensory input, a prediction error signal then allows sensory-motor updates and corrective motor commands. In this framework, it has been proposed that attenuation of neural response observed in fMRI-adaptation studies reveals reduced prediction errors [17], [18]. In the above-mentioned fMRI adaptation studies, repeated manual actions might have caused gradual sensory-motor adaptive learning in posterior parietal and inferior frontal/premotor areas, with reduced prediction errors reflected in BOLD suppression.

Although action goal coding and forward motor-to-sensory control processes have been extensively studied in the context of limb/hand movements, feed-forward control is also a central idea in speech production research (for a recent review, see [23]). For instance, in the DIVA model of speech production (Directions Into Velocities of Articulators; e.g., [24], [25]), modulated responses within the auditory and somatosensory cortices are thought to reflect online adaptive corrective control mechanisms in which auditory and somatosensory consequences are estimated internally from the efference copy of planned motor commands. The auditory and somatosensory consequences are then evaluated with actual sensory input in order to further control production. More specifically, in the DIVA model, the production of a speech sound starts in a ‘speech sound map’, located in the left ventral premotor cortex and the posterior inferior frontal gyrus. Direct feedforward motor commands are sent to the primary motor cortex and the cerebellum. A feedforward control system, composed of an auditory error map (located in the Heschl gyrus and the posterior part of the superior temporal gyrus) and a somatosensory map (located in the ventral somatosensory cortex and the supramarginal gyrus), allows to compare predicted and actual feedback. In case of error detection, corrective motor commands are sent by the right ventral premotor cortex and the posterior inferior frontal gyrus to the primary motor cortex (for similar models derived from state feedback control theory and internal forward model of speech production, see also [26], [27], [28].

The existence of motor-to-somatosensory control loops during silent orofacial movements, devoid of auditory and visual feedback, remains however unclear. Previous studies on simple supralaryngeal lip, tongue and/or jaw movements have provided evidence for a core neural network involved in orofacial motor control as well as “an overall picture of somatotopy with overlap” [29], [30]. In our best knowledge, no study however has attempted to determine the neural correlates of motor-to-somatosensory adaptation during repeated silent orofacial movements. In a previous fMRI study, we determined the core neural network involved in lip, tongue and jaw movements as well as a sensorimotor organization of orofacial articulators [30]. To further extend the above-mentioned results and using an adaptation paradigm, the present fMRI study aims at investigating whether repeated intransitive silent orofacial actions also induce RS in parietal and premotor areas. To this aim, lip protrusion, jaw lowering or tongue retraction movements were repeatedly performed in trains of six consecutive trials, with a sparse-sampling acquisition method used to minimize movement-related image artifacts.

## Methods

### Participants

Eleven healthy adults (nine males and two females with a mean age of 29 years±6), native French speakers, participated in the study after giving their informed consent. All were right-handed according to standard handedness inventory [31], had normal or corrected-to-normal vision and reported no history of motor, speaking or hearing disorders. Participants were screened for neurological, psychiatric, other possible medical problems and contraindications to MRI. The protocol was approved by the Grenoble University Ethical Committee and was carried out in accordance with the ethical standards of the 1964 Declaration of Helsinki.

### Tasks

Three orofacial motor tasks were performed independently and without phonation: a lip protrusion movement, a tongue retraction movement (the tongue turned in the back of the mouth) and a jaw lowering movement. A resting condition, without any movement, served as baseline. For all motor conditions, participants were instructed to initiate and end each movement from a resting state position, with the mouth closed and the tongue and jaw relaxed. In each trial, a 1000 ms visual instruction informed the participants about the motor condition ( “tongue”, “lip”, “jaw”) or the resting baseline (“pause”) and indicated the onset and offset of the movement. Participants were instructed to initiate each motor task as soon as they perceived the visual instruction and to maintain the movement until the visual cue disappeared. Apart from articulatory movements, participants were instructed not to move during the whole experimental session to avoid head-movement artifacts. They were trained a few days prior to the scanning session and all the motor tasks were practiced again just before entering into the scanner. No participant reported any difficulty performing the tasks. This procedure was similar to the one used in Grabski et al.'s study (2012. [30]), except that the motor or resting conditions were here performed in sets of six consecutive trials in order to investigate RS.

### Data acquisition

Magnetic resonance images were acquired with a 3T whole-body MRI scanner (Bruker Medspec S300) with a transmit/receive quadrature head coil. Participants laid supine in the scanner with head movements minimized with a standard birdcage and foam cushions. Visual instructions were presented using the Presentation software (Neurobehavioral Systems, Albany, USA) and displayed on a screen situated behind the scanner and viewed on a mirror fixed above the subject's eyes.

The fMRI experiment consisted of one functional run and one anatomical run. Functional images were obtained using a T2*-weighted, echoplanar imaging (EPI) sequence with whole-brain coverage (TR = 10 s, acquisition time = 2600 ms, TE = 30 ms, flip angle = 90°). Each functional scan comprised forty axial slices parallel to the anteroposterior commisural plane acquired in interleaved order (72×72 matrix; field of view: 216 mm; 3×3 mm2 in plane resolution with a slice thickness of 3 mm without gap). A high-resolution T1-weighted whole-brain structural image was acquired for each participant after the third functional run (MP-RAGE, sagittal volume of 256×224×176 mm3 with a 1 mm isotropic resolution, inversion time = 900 ms, two segments, segment repetition time = 2500 ms, segment duration = 1795 ms, TR/TE = 16/5 in ms with 35% partial echo, flip angle = 30°).

In order to avoid movement artefacts, a “sparse sampling” acquisition paradigm was used (e.g., [32], [33]). This acquisition technique is based on neurophysiological properties of the slowly rising hemodynamic response, which is estimated to occur with a 4–6 s delay in case of speech production (e.g., [30]). In the present study, functional scanning therefore occurs only during a fraction of the TR, alternating with silent interscanning periods, where participants produced orofacial movements. The time interval between the visual instruction onset and the midpoint of the following functional scan acquisition was varied between 4 s, 5 s or 6 s, with the order counterbalanced across trials and conditions.

The motor or resting conditions were performed in three sets of six consecutive trials in a pseudorandom sequence. This RS structure allows measuring changes in BOLD signal for repeated compared to novel performed actions. Altogether, 72 functional scans were therefore acquired (3 motor+1 baseline condition×3 sets×6 repeated trials). In addition, three “dummy” scans at the beginning of each run were added to allow for equilibration of the MRI signal and were removed from the analyses.

### Data analysis

Data were analyzed using the SPM5 software package (Wellcome Department of Imaging Neuroscience, Institute of Neurology, London, UK) running on Matlab 7.1 (Mathworks, Natick, MA, USA). Brain activated regions were labeled using the SPM Anatomy toolbox [34] and, when necessary, using the Talairach Daemon software [35]. For visualization, activation maps were superimposed on a standard brain template using the MRICRON software (http://www.sph.sc.edu/comd/rorden/mricron/). In both group analyses, all activation peaks were first determined in each cluster. The location of maximum activation peaks were then labeled according to probabilistic cytoarchitectonic maps [34] as implemented in the SPM Anatomy toolbox. If a brain region was assigned with a probability lower than 50% or if it was not specified in the SPM Anatomy toolbox, the coordinates of the activation peak was converted from MNI space to the standard stereotactic space of Talairach and Tournoux (1988. [36]) and the related brain region determined using the Talairach Daemon software [35]. With this procedure, the maximum activation peak observed in each anatomical region of each cluster was determined.

#### Data preprocessing

The first three volumes (‘dummy’ scans) were discarded. For each participant, the functional series were first realigned by estimating the 6 movement parameters of a rigid-body transformation in order to control for head movements between scans. After segmentation of the T1 structural image (using the unified segmentation model, [37]) and coregistration to the mean functional image, all functional images were spatially normalized into standard stereotaxic space of the Montreal Neurological Institute (MNI) using segmentation parameters of the T1 structural image. All functional images were then smoothed using a 6 mm full-width at half maximum Gaussian kernel, in order to improve the signal-to-noise ratio and to compensate for the anatomical variability among individual brains.

#### Orofacial action analysis

A first group analysis was performed to determine the neural correlates of each motor task, irrespective of the repetitions. For each participant, neural activations related to the motor tasks were analyzed using the General Linear Model (GLM; [38]), including regressors of interest related to the three motor tasks (lip, tongue and jaw conditions) and realignment parameters, with the silent trials forming an implicit baseline. The BOLD response for each event was modeled using a single-bin finite impulse response (FIR) basis function spanning the time of acquisition (2.6 s). Before estimation, a high-pass filtering with a cutoff period of 128 s was applied. Beta weights associated with the modelled FIR responses were then computed to fit the observed BOLD signal time course in each voxel for each condition. Individual statistical maps were calculated for each motor repetition with the related baseline and subsequently used for group statistics. In order to draw population-based inferences [39], a second-level random effect group analysis was carried-out. A one-way repeated measures analysis of variance (ANOVA) was performed, with the motor condition (3 levels: tongue, jaw, lip) as within-subject factor and the subjects treated as a random factor. Three t-contrasts were calculated first to determine brain regions specifically activated for each of the three orofacial movements compared to the resting condition (p<.05 family-wise-error, FWE, corrected at the voxel level, cluster extent of at least 30 voxels). To identify overlapping activation for all motor tasks, a conjunction analysis [40], [41] was subsequently conducted (p<.05 FWE corrected at the voxel level, cluster extent of at least 30 voxels). Finally, six t-contrasts were calculated to determine brain regions that showed significant change in activity between the motor tasks (tongue>lips, tongue>jaw, lips>tongue, lips>jaw, jaw>tongue, jaw>lips; p<.05 corrected at the cluster level, p<.001 uncorrected at the voxel level, cluster extent of at least 30 voxels).

#### Repetition Suppression analysis

A second group analysis was performed to determine possible RS effects across the 6 consecutive productions over all types of orofacial actions. For each participant, a GLM included 6 regressors of interest (one for each repetition irrespective of the orofacial movement) and 6 realignment parameters, with the silent trials forming an implicit baseline. Individual statistical maps were calculated for each motor repetition with the related baseline and subsequently used for group statistics. A one-way repeated measures analysis of variance (ANOVA) was performed with the “repetition” condition (6 levels: RS1 to RS6) as within-subject factor and the subjects treated as a random factor. Six t-contrasts were first calculated to determine brain regions specifically activated for each of the six consecutive actions compared to the resting condition (p<0.05 FWE corrected at the voxel level, cluster extent of at least 30 voxels).

Three different time-courses of adaptation across the six repetitions were tested, corresponding to a linear decrease, an exponential decrease and a categorical decrease (from the first trial versus the others) of the BOLD response (see [42]). The time courses of adaptation were entered as contrast weights in three parametric T-contrasts (linear decrease: 1 0.6 0.2 −0.2 −0.6 −1; exponential decrease: 1 0.14 −0.17 −0.29 −0.33 −0.35; categorical decrease: 1 −02 −0.2 −0.2 −0.2 −0.2) in order to test the predicted parametric patterns of decreasing BOLD signal amplitude (p<.05 corrected at the cluster level, p<.001 uncorrected at the voxel level, cluster extent of at least 30 voxels). In addition, we tested lateralization of brain activity for linear RS by contrasting individual contrast images with their flipped counterparts (p<.05 corrected at the cluster level, p<.001 uncorrected at the voxel level, cluster extent of at least 30 voxels).

## Results

### Orofacial actions

**Figure 1:**
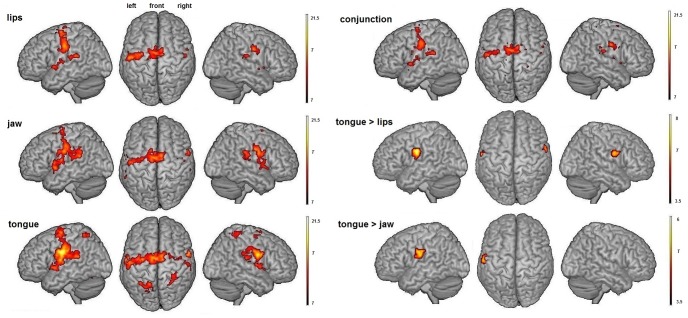
Orofacial actions. Surface rendering of brain regions activated during lip, jaw and tongue movements (left, p<.05 FWE corrected at the voxel level, cluster extent threshold of 30 voxels), showing overlapping activity between the three motor tasks (conjunction, right/top, p<.05 FWE corrected at the voxel level, cluster extent threshold of 30 voxels, see Table 1 for details) and showing significant change in activity between the motor tasks (main effect, right/bottom, p<.05 corrected at the cluster level, p<.001 uncorrected at the voxel level, cluster extent threshold of 30 voxels, see Table 2 for details).

Surface rendering of brain activity and maximum activation peaks observed for the three motor tasks, the conjunction analysis and differences between tasks are provided in Figure 1 and Tables 1 and 2.

**Table 1:** Orofacial actions – similar activations between tasks.

Regions	H	BA	MNI coordinates			T
			x	y	z	
Cluster 1 (519 voxels)						
Supplementary motor area	L	6	−6	−6	56	13.42
Supplementary motor area	R	6	8	−8	58	12.91
Paracentral lobule	L	6	−14	−12	74	7.45
Cluster 2 (457 voxels)						
Primary motor cortex	L	4	−44	−16	38	12.04
Middle frontal gyrus	L	6	−30	−8	68	10.34
Inferior parietal cortex	L	40	−54	−22	34	9.68
Ventral premotor cortex	L	6	−56	−4	32	8.89
Dorsal premotor cortex	L	6	−46	−10	52	8.83
Cluster 3 (267 voxels)						
Primary motor cortex	R	4	50	−8	34	12.61
Ventral premotor cortex	R	6	38	−12	38	11.62
Prefrontal cortex	R	9	54	0	26	7.92
Cluster 4 (198 voxels)						
Insula	L	13	−44	0	2	10.79
Premotor cortex	L	6	−48	−4	8	9.15
Parietal operculum	L	43	−60	−6	14	8.97
Cluster 5 (172 voxels)						
Insula	L	13	−52	−40	20	11.01
Inferior parietal cortex	L	40	−54	−36	22	10.59
Transverse temporal gyrus	L	41	−36	−26	8	10.05
Parietal operculum	L	43	−46	−30	18	8.48
Cluster 6 (115 voxels)						
Claustrum	L		−32	−4	−8	11.19
Putamen	L		−24	−6	−4	9.96
Cluster 7 (61 voxels						
Insula	R	13	46	6	0	10.99
Cluster 8 (38 voxels)						
Parietal operculum	R	43	60	−22	22	9.45
Cluster 9 (33 voxels)						
Cerebellum (declive)	L		−18	−60	−22	9.33

Maximum activation peak summary of brain regions showing overlapping activity between the three motor tasks (conjunction analysis, p<.05, FWE corrected at the voxel level, cluster extent threshold of 30 voxels).

**Table 2:** Orofacial actions – different activations between tasks.

Regions	*p*	H	BA	MNI coordinates			T
	cluster			x	y	z	
**Tongue>Lips**							
Cluster 1 (238 voxels)	**0.000**						
Primary somatosensory cortex		L	3	−56	−10	28	8.02
Primary motor cortex		L	4	−52	−4	20	4.35
Parietal operculum		L	43	−60	−8	14	3.93
Cluster 2 (134 voxels)	**0.004**						
Primary motor cortex		R	4	54	−2	24	5.91
Prefontal cortex		R	9	50	−2	26	5.83
Premotor cortex		R	6	62	0	24	4.70
**Tongue>Jaw**							
Cluster 1 (248 voxels)	**0.000**						
Primary somatosensory cortex		L	3	−56	−10	28	6.17
Parietal operculum		L	43	−60	−12	24	6.07
Inferior parietal cortex/primary		L	3	−62	−16	30	5.20
somatosensory cortex							
Primary motor cortex		L	4	−52	−4	20	4.31
Inferior parietal cortex/primary		L	2	−56	−20	32	4.27
somatosensory cortex							

Maximum activation peak summary and contrast estimates of brain regions showing significant change in activity between the three motor tasks (p<.05 corrected at the cluster level, p<.001 uncorrected at the voxel level, cluster extent threshold of 30 voxels).

Results from the group analysis showed regions that were largely overlapping across the three motor tasks, with the conjunction analysis revealing a bilateral set of common brain areas classically involved in orofacial motor control (see Figure 1 and Table 1). This ‘minimal motor network’ for the three orofacial motor tasks [30] concerned, bilaterally, the activation of the central sulcus extending rostrally onto the precentral gyrus and caudally onto the postcentral gyrus. These two clusters of activations enclosed the superior portion of the ventral premotor cortex (vPM), extending to the dorsal premotor cortex (dPM) in the left hemisphere, the primary motor and somatosensory cortices (with the maximum activation peak located in the primary motor cortex). Bilateral activations were also observed in the supplementary motor area (SMA) as well as in the anterior insular cortex and the parietal operculum. Activity restricted to the left hemisphere was observed in the ventral inferior parietal cortex, extending to the adjacent posterior insular cortex and transverse temporal gyrus, and in the claustrum. Additional left activations were also observed in the dorsal striatum of basal ganglia (putamen) and in the superior part of the cerebellum (declive region of neocerebellum).

In addition, significant changes in activity between the motor tasks were observed (see Table 2). Higher activity was observed for tongue compared to lip movements in the primary sensory-motor cortices, extending to the left parietal operculum and the right premotor/prefrontal cortex, as well as for tongue compared to jaw movements in the left primary sensory-motor cortex, extending to the adjacent parietal operculum and ventral inferior parietal lobule. No significant differences were observed for lip compared to jaw movements.

### Repetition Suppression

**Figure 2:**
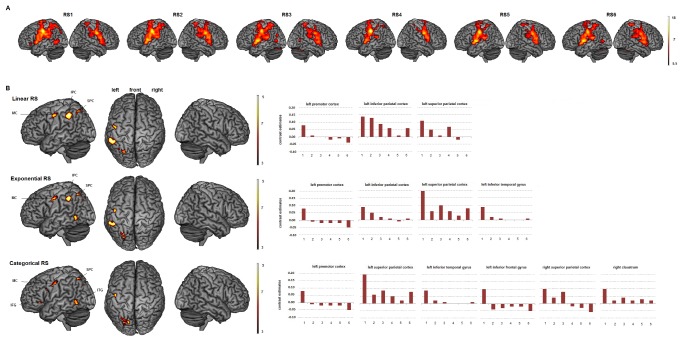
Repetition suppression effect. Up: Surface rendering of brain regions activated in the 6 consecutive productions over all types of orofacial actions (RS1 to RS6) random-effect group analysis, p<.05, FWE corrected, cluster extent threshold of 30 voxels). Down: Brain regions showing linear, exponential and categorical RS and related contrast estimates for the maximum activation peak in each cluster, reflecting percentage BOLD signal change from baseline for the six consecutive performed actions (random-effect group analysis, t-contrast, p<.05 corrected at the cluster level, p<.001 uncorrected at the voxel level, cluster extent threshold of 30 voxels, error bars represent SEM, MC: motor cortex, IPC: inferior parietal cortex, SPC: superior parietal cortex, IFG, inferior frontal gyrus, ITG: inferior temporal gyrus, see Tables 3, 4 and 5 for details).

Surface rendering of brain activity and maximum activation peaks observed in the RS analysis are provided in Figure 2 and Tables 3, 4, and 5.

**Table 3:** Linear repetition suppression analysis.

Regions	*p*	H	BA	MNI coordinates			T	Contrast estimates					
	cluster			x	y	z		1	2	3	4	5	6
Cluster 1 (271 voxels)	**0.000**												
Inferior parietal cortex		L	40	−58	−38	42	5.15	0.14	0.13	0.09	0.06	0.01	0.06
Intraparietal sulcus		L	40	−40	−42	36	4.44	0.11	0.08	0.05	0.03	0.02	0.03
Cluster 2 (173 voxels)	**0.004**												
Ventral premotor cortex		L	6	−34	2	26	4.50	0.08	0.01	0.00	−0.02	−0.01	−0.04
Cluster 3 (108 voxels)	**0.042**												
Superior parietal cortex		L	7	−28	−64	54	4.08	0.11	0.05	0.01	0.07	−0.02	0.00
Inferior parietal cortex		L	40	−32	−50	44	3.74	0.15	0.07	0.04	0.06	0.03	0.06

Maximum activation peak summary and contrast estimates of brain regions showing a linear, RS effect and related contrast estimates reflecting percentage BOLD signal change from baseline for the six consecutive performed actions (random-effect group analysis, t-contrast, p<.05 corrected at the cluster level, p<.001 uncorrected, cluster extent threshold of 30 voxels).

**Table 4:** Exponential repetition suppression analysis.

Regions	*p*	H	BA	MNI coordinates			T	Contrast estimates					
	cluster			x	y	z		1	2	3	4	5	6
Cluster 1 (416 voxels)	**0.000**												
Superior parietal cortex		L	7	−26	−72	38	4.77	0.20	0.06	0.10	0.06	0.03	0.08
Inferior parietal cortex		L	40	−32	−50	44	4.66	0.15	0.07	0.04	0.06	0.03	0.06
Cluster 2 (273 voxels)	**0.000**												
Ventral premotor cortex		L	6	−36	2	26	4.72	0.08	−0.01	−0.02	−0.02	−0.02	−0.05
Inferior frontal gyrus		L	44/9	−42	2	22	4.34	0.09	0.04	0.02	0.03	0.03	0.02
Cluster 3 (223 voxels)	**0.000**												
Inferior parietal cortex		L	40	−50	−42	46	4.89	0.09	0.05	0.02	0.01	−0.01	0.01
Cluster 4 (124 voxels)	**0.001**												
Inferior temporal gyrus		L	19	−46	−56	−6	4.80	0.09	0.02	0.01	0.00	0.00	0.01
Fusiform gyrus		L	37	−52	−56	0	3.99	0.13	0.07	0.06	0.04	0.02	0.06

Maximum activation peak summary and contrast estimates of brain regions showing an exponential RS effect and related contrast estimates reflecting percentage BOLD signal change from baseline for the six consecutive performed actions (random-effect group analysis, t-contrast, p<.05 corrected at the cluster level, p<.001 uncorrected, cluster extent threshold of 30 voxels).

**Table 5:** Categorical repetition suppression analysis.

Regions	*p*	H	BA	MNI coordinates			T	Contrast estimates					
	cluster			x	y	z		1	2	3	4	5	6
Cluster 1 (515 voxels)	**0.000**												
Superior parietal cortex		L	7	−26	−72	38	5.05	0.20	0.06	0.09	0.05	0.02	0.08
Inferior parietal cortex		L	40	−32	−52	44	4.80	0.15	0.06	0.03	0.06	0.03	0.06
Cluster 2 (295 voxels)	**0.000**												
Ventral premotor cortex		L	6	−36	2	26	4.72	0.08	−0.01	−0.02	−0.02	−0.02	−0.05
Premotor cortex		L	6	−40	−2	36	4.62	0.08	−0.01	−0.02	−0.02	−0.02	−0.06
Inferior frontal gyrus		L	44/9	−40	2	34	4.52	0.05	−0.06	−0.03	−0.04	−0.06	−0.08
Primary motor cortex		L	4	−46	−10	46	4.27	0.14	0.09	0.05	0.10	0.07	0.05
Cluster 3 (160 voxels)	**0.000**												
Inferior frontal gyrus		L	47	−34	28	2	4.68	0.10	−0.04	−0.03	−0.02	−0.02	−0.05
Cluster 4 (153 voxels)	**0.000**												
Superior parietal cortex		R	7	8	−68	30	4.31	0.10	0.04	0.08	−0.02	−0.03	−0.06
Cingulate cortex		R	30	26	−64	38	3.38	0.07	−0.02	0.01	−0.01	−0.02	−0.03
Cluster 5 (107 voxels)	**0.002**												
Inferior temporal gyrus		L	19	−46	−56	−6	4.86	0.09	0.02	0.01	0.00	0.00	0.01
Fusiform gyrus		L	37	−44	−58	−12	3.74	0.10	0.02	0.00	0.01	−0.03	0.00
Cluster 6 (107 voxels)	**0.002**												
Claustrum		R		26	18	−6	4.37	0.10	0.02	0.04	0.02	0.03	0.02
Inferior frontal gyrus		R	47	30	22	−14	4.01	0.08	−0.02	0.02	−0.03	0.01	−0.01

Maximum activation peak summary and contrast estimates of brain regions showing a categorical RS effect and related contrast estimates reflecting percentage BOLD signal change from baseline for the six consecutive performed actions (random-effect group analysis, t-contrast, p<.05 corrected at the cluster level, p<.001 uncorrected, cluster extent threshold of 30 voxels).

The use of the repetition suppression paradigm allowed us to determine brain regions sensitive to sensory-motor adaptation, among all brain regions classically involved in orofacial motor control. Linear RS across the 6 consecutive productions over all types of orofacial actions was observed in the left intraparietal sulcus (IPS) and adjacent anterior dorsal IPL, the left superior parietal lobule (SPL, precuneus) extending to the posterior dorsal IPL, and in the precentral and postcentral gyri (with the maximum activation peak for this cluster located in the most dorsal part of the left vPM; see Table 3). Results show a significant lateralization of linear RS only in the left premotor cortex (cluster of 90 voxels, activation peak: x = −36, y = −12, z = 50, T = 5.67, p = .007 corrected). In addition to the premotor and parietal regions showing linear RS, an exponential decrease of BOLD activity was also observed in the left fusiform gyrus and adjacent inferior temporal gyrus as well as in the left posterior inferior frontal gyrus (see Table 4). Finally, except the left intraparietal sulcus and the adjacent anterior dorsal inferior parietal lobule, all the above-mentioned regions were sensitive to categorical decay with additional activity decrease in the triangular part of the inferior frontal gyrus, in the left primary motor cortex, in the right superior parietal cortex and posterior cingulate cortex (see Table 5).

## Discussion

Using sparse temporal acquisition, the goal of this fMRI-adaptation was to investigate RS during repeated intransitive silent lip, tongue and jaw movements. Irrespective of RS, orofacial movements activated a set of largely overlapping, common brain areas forming a core neural network classically involved in orofacial motor control. Crucially, suppressed neural responses during repeated orofacial actions were observed in the left ventral premotor cortex, the intraparietal sulcus, the inferior parietal lobule and the superior parietal lobule. Because lip, tongue and jaw movements were devoid of visual and auditory feedback, these results strongly suggest motor-to-somatosensory adaptive changes during repeated orofacial actions in posterior parietal and ventral premotor cortices.

### Orofacial actions

As provided by the conjunction analysis and apart from RS (see Figure 1 and Table 1), the lip, tongue and jaw movements all activated a set of largely overlapping brain areas, including the sensory-motor and premotor cortices, the supplementary motor area, the parietal operculum and the adjacent left inferior parietal lobule, the insular cortex, the left basal ganglia and claustrum and the left cerebellum. These results appear fully consistent with previous fMRI studies on orofacial motor control, with the above-mentioned brain areas classically assigned to motor preparation, execution and regulation loops [30], [43], [44], [45], [46]. Notably, these results replicate and support findings of a core orofacial motor network observed in a previous study on both supralaryngeal and laryngeal movements, using an identical experimental paradigm but without motor adaptation [30]. In addition, despite large overlap of cerebral activations in all motor tasks, higher activity was observed for tongue compared to lip and jaw movements in primary sensory-motor cortices and right adjacent premotor as well as left inferior parietal regions, likely reflecting more complex motor demands and possibly indicating the motor somatotopy for the tongue. Previous studies indeed demonstrated larger cluster sizes and higher activity for tongue compared to lip and/or jaw movements [30], [44], [46], [47], as well as a similar dorsal localization in the sensory-motor cortex for tongue movements (for a review, see [30]).

### Repetition Suppression

Crucially, linear and exponential RS across the six consecutive productions over all types of orofacial actions were observed in the left vPM, the IPS and adjacent antero-dorsal IPL, as well as the SPL extending to the postero-dorsal IPL (see Figure 2 and Tables 3, 4 and 5). A lateralization analysis on linear RS further showed a significant RS lateralization within the left premotor cortex, a result in line with the DIVA model and a left lateralization of the premotor cortex in speech production [24], [25]. Interestingly, the left fusiform gyrus and adjacent inferior temporal gyrus as well as the inferior frontal gyrus were activated in the first trial but not in the others (as observed in the exponential and categorical RS analyses). This result likely reflects visual and lexical processing of orthographic instructions in the first trial [10]. The cingulate cortex also showed categorical activity decrease from the first trial to the others. Due to its connections with prefrontal and parietal cortices, this region is known to play a role in higher-order motor control functions such as motor attention and movement selection [48] and has been previously reported to be activated during simple [30] and complex [49] orofacial movements. Finally, activity in the left IPS and the adjacent antero-dorsal IPL decreased in a step-wise fashion (as revealed by the linear and exponential analyses but not the categorical analysis).

Altogether, these results appear exquisitely in line with previous studies on manual action goal coding in both nonhuman and human primates. As previously mentioned, neurophysiological recordings of single neurons in nonhuman primate have shown neuronal selectivity for action goal coding of transitive manual motor acts in posterior parietal and ventral premotor areas [1], [2], [3], [4], [5], [6], [7]. In humans, evidence for action goal coding in fronto-parietal areas first comes from the pioneering work of Liepmann [50] and studies on ideomotor apraxic patients. These patients are characterized by the inability to correctly imitate hand gestures and voluntarily pantomime tool use, mostly associated with lesions located in the left intraparietal sulcus and premotor areas (e.g., [51]), despite adequate strength, dexterity, and comprehension. Previous fMRI-adaptation studies on humans also revealed that repeated transitive and communicative intransitive manual actions with similar goal cause RS in the anterior intraparietal sulcus as well as in the inferior frontal gyrus and the premotor cortex [8], [10], [11], [16]. Interestingly, these observed RS in posterior parietal and premotor areas are not restricted to motor acts but also appeared during the repeated observation of similar actions [8], [9], [11], [12] as well as during a cross-modal paradigm, with suppressed responses when manual actions were first observed and then executed and vice versa [11]. Although debated, RS during both executed and observed actions in this fronto-parietal circuit has been largely discussed in the context of action understanding and the human mirror-neuron system (e.g., [11], [12]). However, because we here only focused on motor control, this question is out of the scope of the present study.

As in the present study, although parietal and premotor areas were the most consistent regions showing RS in previous fMRI-adaptation studies on repeated motor acts, suppressed response in the SPL has also been observed during repeated manual actions [8], [9], [10], [12]. It has been suggested that the SPL would be mainly involved in low-level action representations for visuomotor transformation while the IPL would represent higher-level representations for action goal coding [52]. Since no visual feedback were of course provided during repeated orofacial actions and because of the potential contribution of SPL to visuospatial processing, one possible interpretation of RS in the left SPL would be related to visuospatial imagery of orofacial movements. Although speculative, this hypothesis gained support from a previous fMRI study on spatial processing of tongue movement [53]. In this study, participants were asked to perform either a tongue movement precisely directed to the left or right, upper or lower, incisor, canine, or molar tooth or to do a tongue retraction. Stronger SPL, IPL and premotor activations were observed during the former movements compared to tongue retraction. These results might therefore indicate stronger processing demands for both tongue-centered coordinate coding of the tooth target and action-goal coding. Interestingly, a double dissociation between motor and visual Imagery was also observed between IPL and SPL using repetitive transcranial magnetic stimulation [54], with IPL virtual lesions selectively altering motor imagery and motor representations of movements whereas SPL lesion only affected visual imagery and visuospatial representations.

Given that the left ventral premotor cortex, the intraparietal sulcus, the inferior parietal lobule and the superior parietal lobule have been previously shown to be sensitive to RS during repeated produced and/or observed manual actions in both nonhuman and human primates, these findings further extend the role of these regions in action goal coding and forward sensory-motor control to silent intransitive orofacial actions. Since no visual or auditory feedback occurred during orofacial actions, this likely suggests a supramodal function of these regions in forward predictive sensory-motor control, being visuo-motor and/or somatosensory-motor. As previously mentioned, while action goal coding and forward sensory-motor control processes have been extensively studied in the context of upper limb movements, generative forward models of speech production also argue for a role of the auditory and somatosensory cortices in online predictive/corrective control mechanisms in which sensory consequence of the speech motor act are evaluated with actual sensory input in order to further control production (e.g., [24], [25], [26], [27], [28]. It is indeed well established that auditory feedback plays an important role in tuning the speech motor control system. For instance, transient, unexpected transformations of the acoustic consequences of speaking lead to on-line and rapid articulatory adjustments (e.g., [55], [56]) and perceptuo-motor adaptation even when the perceptual manipulation is removed (e.g., [57], [58]; At the neural level, increased activity has also been observed in the auditory cortex during altered or delayed auditory feedback (e.g., [59], [60], [61], [62] as compared to normal auditory feedback. Although somatosensory information is a less recognized source of sensory input accompanying speech production and vocalization, previous behavioral studies demonstrated rapid motor changes during unexpected orofacial mechanical perturbations or by anesthetizing the vocal folds (e.g., [63], [64], [65], [66], [67], [68]). In the present study, the use of the repetition suppression paradigm allowed to determine brain regions sensitive to sensory-motor adaptation, among all brain regions classically involved in orofacial motor control. The present results thus complement these latter findings by highlighting a role of the left ventral premotor and posterior parietal brain areas in forward motor-to-somatosensory control during silent orofacial movements. Previous fMRI studies have underlined largely overlapping brain areas during the production of speech and non-speech vocal tract gestures (e.g., [30], [69]), notably in the the premotor and parietal cortices. Because orofacial actions were here devoid of auditory feedback, our results support gradual somatosensory adaptive learning and reduced prediction errors in posterior parietal cortices, as suggested in recent neurobiological models of speech production [24], [25], [26], [27], [28].

### Limitations

Despite strong evidence for RS in left frontal and parietal areas, some methodological issues which could impact on the interpretation of the data have to be finally addressed. First, force parameters were not monitored during the scanning session, we therefore cannot exclude that the observed RS might partly derive from force decrease or muscle fatigue during repeated movements. From this possibility, a previous fMRI study on hand muscle fatigue provided evidence for BOLD decrease in the precuneus [70], this effect being due for the authors to sustained muscle contraction and related increased arterial blood pressure [71]. In contrast, muscle fatigue classically involves an increase of BOLD response over time in sensory-motor areas (e.g., [70], [72], an effect most probably due to motor compensation driving mechanisms in order to maintain performance. Also, as to force parameters, kinematic data were not collected during scanning. Since normal force and kinematic movement variability certainly occurred from trial to trial, our results likely suggest that the observed RS in the fronto-parietal circuit does not depend on precise replication of an action from one trial to another but rather on action goal coding. A second important methodological issue is that no separate RS analysis for each articulator was performed. Performing independent RS analyses for each orofacial articulator would have required a greater number of trials per motor task and repetition, significantly increasing the duration of the study. One other possibility would have been to limit the number of successive repeated movements. However, in our study, we used six consecutive occurrences performed in distinct trials in order to precisely explore the timing of adaptation effect [42]. For that reason, each of the six repeated movements was modelled irrespectively of the motor task in the RS analysis. Hence, although our results suggest forward motor-to-somatosensory adaptive changes during repeated orofacial actions in premotor and parietal areas, we cannot rule out that the observed RS might be more related to one or the other orofacial movements. From this view, stronger BOLD response was observed for tongue retraction in a region of the left ventral premotor cortex close to the one showing RS. Future studies are therefore required to further and specifically determine RS for each of the tongue, lip and jaw movements.

## Conclusions

This fMRI-adaptation study was designed to determine the neural correlates of action goal coding and forward predictive processes during repeated silent and intransitive lip, tongue and jaw movements. In line with previous studies on manual actions, suppressed neural responses during repeated orofacial actions were specifically observed in the left ventral premotor cortex, the intraparietal sulcus and adjacent antero-dorsal inferior parietal lobule as well as the superior parietal lobule extending to the postero-dorsal inferior parietal lobule. These results provide evidence for motor-to-somatosensory adaptive changes in this fronto-parietal circuit.
